# Optimization of a method for the clinical detection of serum exosomal miR-940 as a potential biomarker of breast cancer

**DOI:** 10.3389/fonc.2022.956167

**Published:** 2022-10-21

**Authors:** Zhiyun Gu, Haojie Yin, Haiwei Zhang, Hui Zhang, Xiaoyu Liu, Xiaohua Zeng, Xiaodong Zheng

**Affiliations:** ^1^ Department of Oncology Laboratory, Chongqing University Cancer Hospital, Chongqing, China; ^2^ Chongqing Key Laboratory of Translational Research for Cancer Metastasis and Individualized Treatment, Chongqing University Cancer Hospital, Chongqing, China; ^3^ Bioengineering College, Chongqing University, Chongqing, China; ^4^ Medical College of Chongqing University, Chongqing University, Chongqing, China

**Keywords:** exosomes, reference genes, miR-940, RT-qPCR, breast cancer

## Abstract

Serum exosomal microRNAs (miRNAs) are potential biomarkers for tumor diagnosis. Clinically, reverse transcription-quantitative polymerase chain reaction (RT−qPCR) can be used to determine the expression of exosomal miRNAs in the serum of breast cancer patients. The prerequisites for obtaining meaningful serum exosomal miRNA data of breast cancer patients include a suitable extraction method for exosomes and RT−qPCR data standardized by internal reference genes. However, the appropriate methods for the extraction of exosomes and the applicability of reference genes for analyzing exosomal miRNAs in breast cancer patients remain to be studied. This study compared the effects of three exosome extraction methods as well as the expression of exosomal miRNA in different initial serum amounts and at different serum states to identify the selection of the best method for serum exosome extraction. Five candidate reference genes including miR-16, miR-484, miR-1228, miR-191 and miR-423 for standardizing serum exosomal miRNAs were screened using five algorithms and were used for the quantification of serum exosomal miR-940. Significant downregulation of serum exosomal miR-940 expression in breast cancer was detected using miR-191 and miR-1228, whereas no significant down or up regulation was observed with miR-484, miR-423 and miR-16. Previous studies have shown that the expression level of miR-940 is downregulated in breast cancer tissues. The absolute quantitative results showed that miR-940 was significantly downregulated in breast cancer serum exosomes, which was consistent with the results from the analysis using miR-191 or miR-1228 as reference genes. Therefore, miR-191 and miR-1228 could serve as reference genes for the relative quantification of serum exosomal miRNAs. This finding indicated the importance of rigorously evaluating the stability of reference genes and standardization for serum exosomal miRNA expression. Moreover, the level of serum exosomal miR-940 in breast cancer could reflect the presence of lymph node metastasis and the status of HER2/neu, which indicates its potential as a biomarker for breast cancer metastasis. In summary, an optimized protocol for the detection of serum exosomal miR-940 as a breast cancer marker was preliminarily established.

## Introduction

MicroRNAs (miRNAs) are short noncoding RNA molecules that can participate in the regulation of posttranscriptional gene expression (1). Such regulation is essential for embryonic development, tumor initiation, immune modulation, and other biological processes. Exosomes are 40-100-nm disc-shaped vesicles. Various types of cells can release exosomes under normal and pathological conditions (2). Exosomes are involved in cell–cell communication and are released from donor cells into the microenvironment to affect target cells. This biological process exerts important biological functions (1). To date, exosomes have been found in various body fluids, such as human blood, urine, tears, breast milk, and semen (3).

An et al. verified that exosomes contain many proteins, cytokines, DNA, mRNAs, miRNAs, lncRNAs, and other nutritional elements (4). A previous study showed that exosomal miRNAs play an important role in communication between breast cancer cells (4, 5). A cohort study compared the serum levels of exosomal miRNAs between healthy women and breast cancer patients with different molecular subtypes and found that exosomal miRNAs can be regarded as blood-specific biomarkers for more aggressive tumors, such as triple-negative and hormone receptor-negative breast cancer (6). Another study noted that exosomes are potential biomarkers for ovarian cancer and breast cancer (7). Zhou et al. proved that the content level of miRNAs is significantly altered in serum exosomes from breast cancer patients, which indicates that exosomal miRNAs can be used as biomarkers for the identification of breast cancer (8). Additionally, Rodriguez et al. proved that the expression of exosomal miRNAs found at high levels in serum can be used as a biomarker of breast cancer (9).

Several different histological subtypes of breast cancer have been identified, and each subtype has different levels of invasiveness, clinical presentations, treatment programs, and prognoses (10). The diagnosis of breast cancer must be made as soon as possible such that treatment can be started in time. The difference in the expression level of exosomal miRNAs between healthy people and breast cancer patients can be used as a biomarker for the prediction of breast cancer.

Accurately measuring the level of miRNA in exosomes is the first step for its detection as a biomarker (11). RT–qPCR is a precise method for detecting and quantifying circulating miRNAs. To ensure that the measurements are comparable across different samples, the same volume of each body fluid is needed for RNA extraction. Despite using the same initial biofluid volume for each sample, the total RNA levels will not be consistent between samples due to different upstream procedures for RT–qPCR, such as sample preparation, exosome isolation, and miRNA extraction. Hemolysis may occur in clinically collected blood samples, and the effect on exosomal RNA is unknown (12). Therefore, standardized sample collection must be performed to address these problems. Notably, stable internal reference genes are essential for qPCR standardization and accurate miRNA quantification. Differences in expression results may not be caused by the disease itself but may instead be caused by differences in the processes used for sample acquisition, sample preservation, RNA extraction, and target gene quantification (13).

Therefore, appropriate methods for exosome extraction and optimal reference genes with stable expression must be determined to accurately normalize the quantitative data of exosomal miRNA (14). To date, in the RT–qPCR analysis of breast cancer exosomal miRNA, a consensus has not been reached regarding appropriate exosome extraction methods and appropriate internal reference genes.

MicroRNA-940 (miR-940) is a recently identified miRNA family member that is abnormally expressed in cardiovascular and neoplastic diseases. This miRNA has different expression levels in different diseases; in gastric cancer, the expression level of miR-940 is increased, whereas in cardiovascular disease, the expression level of miR-940 is decreased (15–17), which indicates its important regulatory role. Previous studies found that triple-negative breast cancer tissues express lower levels of miR-940 compared with normal tissues and that proliferation and migration in triple-negative breast cancer are affected by miR-940 (18). However, the clinicopathological relationship between serum exosomal miR-940 and breast cancer patients is unclear.

To select a suitable methods for serum exosome extraction that can be used in clinical practice, we extracted exosomes from the same sample using three extraction methods, analyzed the expression differences of serum exosomal miRNAs among different methods, analyzed the effect of differences in the initial serum volume on the RT–qPCR results, and finally analyzed the changes in hemolytic blood sample exosomal miRNAs in blood samples and normal blood samples.

Based on previous studies, various candidate reference genes were used to normalize the expression levels of exosomal miRNAs in breast cancer patients and healthy controls to screen the reference genes. A total of 5 candidate internal reference genes were selected based on previous reports of their suitability for RT–qPCR analyses of cancer using tissues or serum. Two of these genes were previously described as reference genes for exosome miRNA analysis: miR-16 and miR-484 (6). The remaining genes, miR-1228, miR-191, and miR-423, were obtained from other studies (19, 20). Five genes were initially screened as internal reference genes of serum exosomal miRNA, and the expression levels of the five candidate genes were then detected. The expression stability of these five genes was evaluated to determine suitable reference genes. The screened internal reference genes were used to evaluate the expression level of miR-940 and verified that the results of the screened internal reference genes were stable and reproducible.

Moreover, the applicable clinical exosomes extraction method identified from our comparison of breast cancer serum and normal serum was used to explore the differential expression of exosomal miR-940 using the selected stable reference genes. The serum exosomal miR-940 copy number and clinical data for breast cancer patients were analyzed by absolute quantification, and the results confirmed that serum exosomal miR-940 could serve as a metastatic marker for breast cancer.

This study provided the first identification of suitable reference genes for the clinical study of serum exosomal miRNA expression in breast cancer while screening pretreatment methods suitable for the clinical extraction of serum exosomes; moreover, this study demonstrated that serum exosomal miR-940 could serve as a potential metastatic marker for breast cancer.

## Materials and methods

### Patient and control sample collection

This study was approved by the Ethics Committee of the Chongqing University Cancer Hospital. Blood samples were donated by the Chongqing University Cancer Hospital. The blood sample set was composed of 118 breast cancer patients and 40 healthy controls. The all blood samples collected from patients were collected before treatment (including radiotherapy, chemotherapy, and surgery). The blood samples from patients without any treatment were drawn on an empty stomach, whereas those from healthy controls were also drawn early in the morning. A total of 5 mL of whole blood was drawn from each donor. Peripheral blood was collected into a tube containing heparin sodium and centrifuged at 3000×g for 10 min. The upper part of the serum was collected into a brand new centrifuge tube and centrifuged at 12000×g for 10 min to remove a cellular component. All centrifugation operations were performed at 4 °C. The separated serum was stored at -80 °C. To standardize the treatment of different serum samples, exosomal RNA was extracted from 1000 µL of serum, and the samples were preliminarily standardized according to a uniform volume. We randomly selected 59 of them to verify the stability of the candidate genes. The remaining 59 cases were used to investigated the relationship between serum exosomal miR-940 expression level and patient physiological indicators, so we looked up the clinicopathological reports of these patients. The clinicopathological were provided by the Department of Pathology and specific clinical data were presented in Schedules 1 and 2 (**Supplementary Table 1**, **Supplementary Table 2**).

### Serum exosome isolation

Exosomes were extracted by ultracentrifugation, membrane affinity, and precipitation. Equal portions of the collected serum were removed from the -80 °C refrigerator, thawed on ice, and centrifuged at 12000×g and 4 °C for 10 min, and 1 mL of serum was then collected. Ultracentrifugation was performed using a 10-mL sample consisting of 1 mL of serum sample and 9 mL of PBS, and the sample was centrifuged by ultracentrifuge (Optima L-100XP, Beckman, USA) at 4°C and 100000×g for 70 min. The supernatant was carefully removed from the ultracentrifugation tube, and the remaining 2 mL of liquid containing exosomes was mixed with 8 mL of PBS. The supernatant was centrifuged again at 100000×g and 4°C for 70 min. Exosomes were resuspended in 200 μL of PBS and stored at -80 °C. Precipitation was performed with ExoQuick exosome precipitation solution (SBIS, System Biosciences, USA). The reagents were added to 1 mL of serum, and the mixture was shaken thoroughly. The flocculent precipitate appeared and was allowed to rest overnight at 4°C. The supernatant was then centrifuged at 12000×g for 10 min, and the precipitate contained exosomes. The exosomes were resuspended in 200 μL of PBS and stored in a -80°C refrigerator. The membrane affinity method was performed by collecting exosomes using an exoEasy Maxi Kit (QIAGEN GmbH, Hilden, Germany). Briefly, buffer XBP and serum were mixed in equal volumes, 2 mL of the mixture of each sample was then added to the exoEasy spin column and centrifuged at 4200×g for 1 min, and the waste after centrifugation was discarded. Ten milliliters of buffer XWP was added to the spin column and centrifuged at 5000×g for 5 min to remove the buffer XWP. Next, 200 μL of Buffer XE was added to the spin column and incubated for 1 min. The XE buffer was collected after centrifugation at 5000×g for 5 min. Exosomes were resuspended in XE buffer and stored at -80 °C.

### Characteristic analysis of exosomes

The extracted exosomes were characterized using different methods. The characteristics of the exosomes were determined by transmission electron microscopy (TEM) (Tecnai G2 F30S-TWIN, FEI, USA). The exosomal particle size was measured using a nanoparticle tracking analysis (NTA) instrument (ZetaView, Particle Metrix, Meerbusch, Germany) according to the experimental requirements. The characteristic proteins of exosomes, such as CD9, CD63, and TSG101, were analyzed by Western blotting.

Monoclonal anti-CD63 (#98327) antibodies, anti-CD9 (#52090) antibodies, and anti-TSG101 antibodies (#72312) were obtained from Cell Signaling Technology (Denver, MA, USA).

### Reference genes and primer design

The sequence of the target gene was obtained from the NCBI and miRBase databases, and the primers were designed according to the requirements of the SYBR Green method for RT–qPCR and were synthesized by Biotechnology (Biotechnology, Shanghai, China). The primer information of each gene designed is shown in **Table 1**.

**Table 1:** Primer information of each gene.

Accession number	Gene	Primer
MIMAT0004983	hsa-miR-940	5’-ATAAGGCAGGGCCCCCGCT-3’
MIMAT0000069	hsa-miR-16-5p	5’-TAGCAGCACGTAAATATTGGCG-3’
MIMAT0002174	hsa-miR-484	5’-TCAGGCTCAGTCCCCTCCCGAT-3’
MIMAT0001340	hsa-miR-423-3p	5’-AGCTCGGTCTGAGGCCCCTCAGT-3’
MIMAT0000440	hsa-miR-191-5p	5’-CAACGGAATCCCAAAAGCAGCTG-3’
MIMAT0005583	hsa-miR-1228-3p	5’-TCACACCTGCCTCGCCCCCC-3’

### RNA extraction and RT–qPCR

The exosomes extracted from each sample were lysed with 1 mL of QIAzol^®^ (Qiagen GmbH, Hilden, Germany). The quality of the RNA was assessed by measuring the A260/A280 value of the extracted RNA using a NanoDrop™ One/OneC (Thermo Scientific, China). These cDNAs were obtained by reverse transcription of 1 ng of the extracted exosomal RNA using a miRcute Plus miRNA first-strand cDNA kit (TianGen, Beijing, China). The thermal cycling parameters for reverse transcription were 60 min at 42 °C and 3 min at 95 °C. The cDNA samples were diluted 10-fold in nuclease-free water and stored at −20 °C.

The expression levels of candidate internal reference genes in the serum exosomes were analyzed by RT–qPCR, which was performed with 384-well reaction plates using a LightCycler 480 II (Roche, Germany). The qPCR conditions are shown in **Table 2**. The data were analyzed using the software provided for the fluorescence quantitative PCR system. When the Ct value is greater than 35, the data have no meaning and are regarded as unexpressed, and this type of data is removed. Moreover, the arithmetic average of the Ct values of the three wells was used as the final Ct value of the miRNA PCR amplification.

**Table 2:** RT−qPCR procedure.

Cycle	Temperature	Time
1X	95°C	15 min
5X	94°C	20 sec
	63~65°C	30 sec
	72°C	34 sec
1X	94°C	20 sec
	60°C	34 sec

### Determination of the miR-940 copy number

The method used for copy number determination was described in the literature (21). Briefly, standard curves for miR-940 expression in serum exosomes were constructed by a serial dilution series of standard miR-940 ranging from 100 to 10^6^ copies/μL. The plasmid copy number was calculated using the following equation:


$$\frac{6.02\times 10^{23}(\text{copy}/\text{mol})\times \text{DNA amount(g})}{\text{DNA length(bp})\times 660(\text{g}/\text{mol}/\text{bp})}$$

The corresponding logarithm template copy number was then plotted against the Ct values obtained by real-time qPCR. Statistical analyses were performed using Student’s t test (two-tailed) to analyze the differences between groups. All values are expressed as the means ± S.E.Ms. A value of P< 0.05 was regarded as statistically significant.

### Statistical analysis

First, the standard deviation (SD) and coefficient of variation (CoV) of each sample were calculated. In addition, four commonly used methods were applied to more comprehensively assess the stability of the candidate genes: geNorm (22), NormFinder (23), BestKeeper (24), and the comparative ΔCt method (25). These four methods use online tools to evaluate reference gene expression (26). Moreover, statistical analyses were performed with the paired t test using SPSS Statistics 21 software, and a P value less than 0.05 indicated significant differences.

## Results

### Characterization of serum exosomes

**Figure 1:**
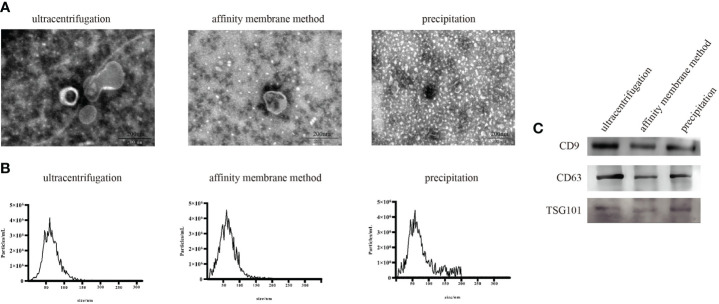
Characterization of exosomes extracted using three exosome extraction methods. **(A)** Physical examination of the isolated exosomes. **(B)** NTA of the isolated exosomes. **(C)** Western blot results of the isolated exosomes.

The first step in the evaluation of exosomal miRNAs is the successful isolation of exosomes from serum. Ultracentrifugation, membrane affinity, and precipitation were used to isolate exosomes from serum, and a physical examination by transmission electron microscopy (TEM) showed a spherical structure of 30~150 nm and a typical doughnut-like shape (**Figure 1A**). The characteristics of the exosomes were consistent with those previously reported (27). Nanoparticle tracking assessment (NTA) analysis showed that the average particle size of exosomes ranged from 110 nm to 140 nm (**Figure 1B**). A Western blot analysis of the characteristic proteins of serum exosomes (3), i.e., CD63, CD9, and TSG101, was performed (**Figure 1C**).

### The selection of the best method for serum exosome extraction

Twenty serum samples were randomly selected from our collected serum samples and divided into two groups to screen the best method for the clinical serum exosome extraction. We analyzed the effects of various exosome extraction methods and the starting amount of serum samples on the expression levels of three serum exosomal miRNAs, analyzed the expression levels of three serum exosomal miRNAs under hemolytic conditions, and established a pretreatment method suitable for the clinical detection of serum exosomal miRNAs.

The results from the literature and our experiments confirmed that all three extraction methods could extract exosomes. Therefore, through the detection of miRNA expression levels in exosomes using three different extraction methods, the influence of different extraction methods on the serum exosomal miRNA levels was analyzed. miR-16, miR-1228 and miR-940 were selected: the first two represent internal reference genes, and the last one represents the target gene of this study.

**Figure 2:**
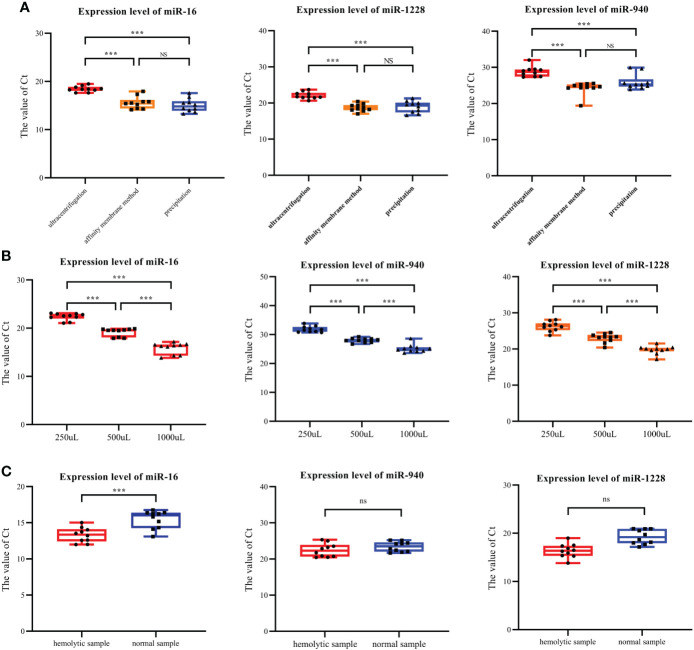
Expression levels of exosomal miR-940, miR-1228 and miR-16 under various conditions. **(A)** The three types of miRNAs were found at the lowest levels in the exosomes isolated by ultracentrifugation. **(B)** Three miRNAs were expressed in serum exosomes obtained with initial serum levels of 250, 500 and 1000 μL. **(C)** Expression of three exosome miRNAs in hemolysis and normal blood samples. (*p<0.05, **p<0.01, ***p<0.001, no significance, ns p>0.05).

Among the three extraction methods, the exosomes extracted by ultrasonication had the lowest content of the three miRNAs tested. No significant differences in the contents of the three miRNAs were identified between exosomes extracted using the membrane affinity method and exosomes obtained using the precipitation method (**Figure 2A**). However, the coefficient of variation (CoV) of miRNA expression in exosomes extracted using precipitation was higher than the coefficient of variation (CoV) obtained for exosomes extracted using membrane affinity (**Table 3**). Therefore, membrane affinity was selected for exosome extraction in all subsequent experiments.

**Table 3:** Coefficient of variation (CoV) of miRNA expression in exosomes extracted using different methods.

	Ultracentrifugation	Affinity membrane method	Precipitation
miR-16	0.031891952	0.079909031	0.091907362
miR-1228	0.04270173	0.054146769	0.083191199
miR-940	0.048764364	0.073463689	0.081203994

RT–qPCR was used to detect the expression levels of miR-16, miR-1228 and miR-940 in the exosomes extracted from the different initial volumes of serum from breast cancer patients. The initial serum levels were 250, 500 and 1000 μL. When the serum volume of breast cancer patients doubled, the Ct values of all miRNAs decreased over three cycles (**Figure 2B**). Therefore, for small blood samples, a serum volume lower than that detailed in the instructions could also be used for exosome extraction.

In the process of preserving samples, hemolysis occurred in 10 samples, and we subsequently collected normal blood samples from these patients. Therefore, we studied the influence of hemolysis on the content of miRNA in the exosomes of the samples. Exosomal RNA was extracted from both hemolytic and normal blood samples, and the same method was used for the extraction of exosomal miRNA from hemolysis samples. The levels of miR-16, miR-1228 and miR-940 were detected. The RT–qPCR results showed that in hemolyzed blood, the expression levels of miR-16, miR-940 and miR-1228 in the extracted exosomes were significantly higher than those in serum exosomes without hemolysis (**Figure 2C**). This finding is likely because blood cells release exosomes during blood storage, which alters the amount of exosomes that will be collected through plasma/serum. In plasma or serum samples, the quantification of exosomal miRNAs may be impaired due to contamination with erythrocyte-derived miRNA caused by hemolysis. Therefore, in clinical serum exosome experiments, hemolysis may increase the expression level of target genes.

### Screening of candidate reference genes and analysis of their applicability

**Figure 3:**
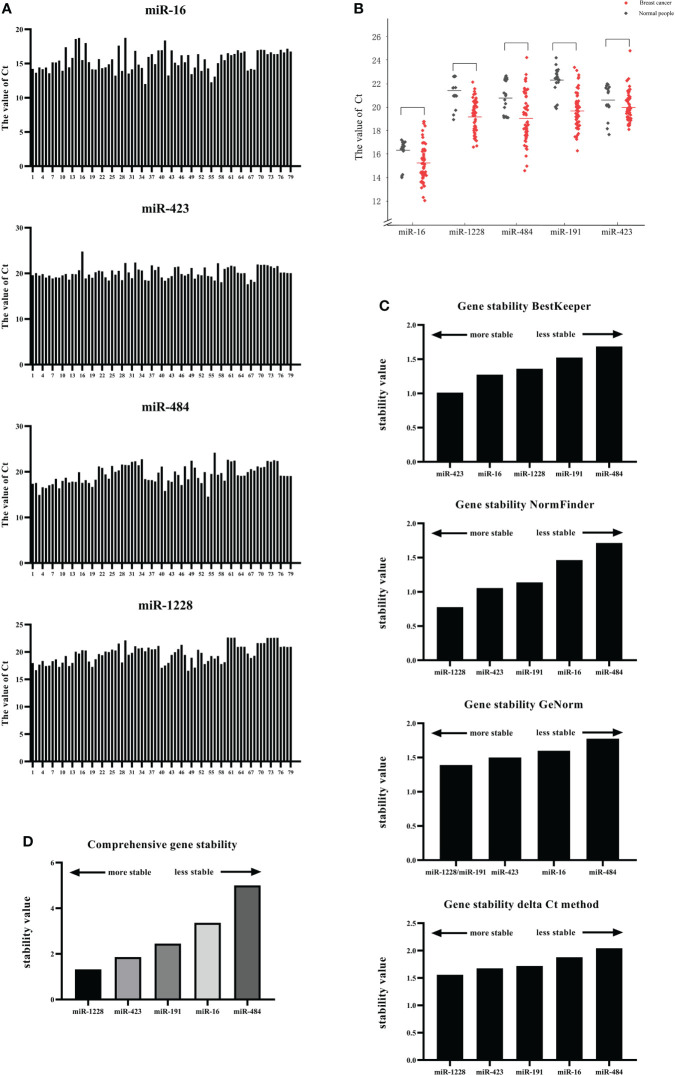
Screening of candidate reference genes and analysis of their applicability. **(A)** Expression of candidate internal reference genes in serum exosomes under breast cancer and healthy conditions. **(B)** The expression levels of miR-16, miR-1228, miR-484, miR-191, and miR-423 in the serum exosomes of breast cancer patients and normal human serum exosomes were compared. **(C)** The stability of the five candidate internal genes was analyzed and sorted using four algorithms, namely, GeNorm, NormFinder, BestKeeper, and ΔCT. **(D)** The stability of the five candidate internal genes was analyzed and sorted using RefFinder.

Because the primary requirement of internal reference genes is to present similar expression levels under diseased and healthy conditions, we compared the expression of candidate internal reference genes in serum exosomes under breast cancer and healthy conditions. We read out the Ct values of different candidate genes in each serum sample (**Figure 3A**). Among them, the serial numbers 1-20 correspond to samples from the controls, whereas the numbers 21-79 correspond to samples from breast cancer patients. The picture shows the Ct value of different candidate genes obtained for each serum sample. The Ct values ranged from 12.01 (miR-16) to 24.78 (miR-432).

Subsequently, the expression levels of miR-16, miR-1228, miR-484, miR-191, and miR-423 in the serum exosomes of breast cancer patients and normal human serum exosomes were compared (**Figure 3B**). The results showed that the Ct value of each candidate gene was not significantly different between the serum exosomes from the breast cancer patients and those from the controls. We also showed the dispersion of the Ct values (**Table 4**).

**Table 4:** Dispersion of the Ct values.

Gene	miR-16	miR-1228	miR-484	miR-191	miR-423
STDEV	1.511947796	1.632827144	2.035326422	2.035326422	1.275306533

The stability of the five candidate internal genes was analyzed and sorted using four algorithms, namely, BestKeeper, NormFinder, GeNorm, and ΔCT; however, the four methods used to analyze the stability of the internal genes were not the same (**Figure 3C**).

BestKeeper compared the correlation coefficient (r), standard deviation (SD), and coefficient of variation (CV) generated by the pairing of each gene and ultimately determined a relatively stable internal reference gene. The principle was that a more stable internal reference gene would have a smaller standard deviation and coefficient of variation and a larger correlation coefficient. Gene stability was also judged according to the SD value. At SD>1, the expression of the internal reference gene was unstable. The results obtained with BestKeeper are shown in **Figure 3C**, which indicates high SD variation among miR-1228, miR-16, and miR-484. In this study, miR-423 was the most stable gene (std dev=1.012), followed by miR-16 (std dev=1.275).

NormFinder software was designed by Claus et al. to screen out internal reference genes suitable for RT−qPCR. A linear scale was used for quantification of the raw data, analyze the stability of candidate genes and provide the stability value of each gene: a higher stability value indicates lower stability of the gene as an internal control and thus indicates that the gene is not suitable as an internal control in this experiment. Among the five candidate genes tested in this study, the results obtained using NormFinder showed that miR-1228 has the lowest stability value of 0.778, which indicates that this gene was the most stable internal reference gene in this experiment, followed by miR-423 (1.056) and miR-191 (1.139), and the least stable gene was miR-484.

The standard for using GeNorm to evaluate the stability of the internal reference genes was to calculate the average coefficient of variation M value of the logarithmic conversion value of the ratio of the first gene to the remaining genes. The M value must be less than 1.5, and a smaller M value corresponds to greater stability of the gene as an internal reference. The final result obtained by calculation was two or more candidate combinations. The results obtained using GeNorm showed that the M values of miR-484, miR-423 and miR-16 in the sample were higher than 1.5, indicating that these candidate genes are unreliable and cannot be used as internal reference genes for the standardization of breast cancer serum exosomal RNA. The most stable gene combination was the combination of miR-1228 and miR-191, which had an expression stability M value of 1.391.

The comparative ΔCt method was used to analyze the stability of the internal reference gene, and the result was a combination of two genes. The ΔCT method could eliminate the influence of coordinated regulation and evaluate the reference genes from various aspects. The results obtained using the ΔCt method indicated that miR-1228 and miR-423 constituted the most stable group.

Because the four analysis methods use different algorithms, the results obtained were also different; thus, normalization and integration of the data were performed when necessary. RefFinder is a web tool that can synthesize the results from the four software programs to generate the final overall ranking of reference genes. According to the output, the most stable reference gene was miR-1228, and the lowest and most unstable reference gene was miR-484 (**Figure 3D**). These results indicated that miR-1228 may be used as the most stable reference gene in breast cancer research.

### Impact of reference genes on the expression levels of target genes

RT–qPCR analysis was applied to further evaluate the stability of each candidate reference gene in the sample. miR-940 exhibits low expression levels in the serum of breast cancer patients (28, 29).

**Figure 4:**
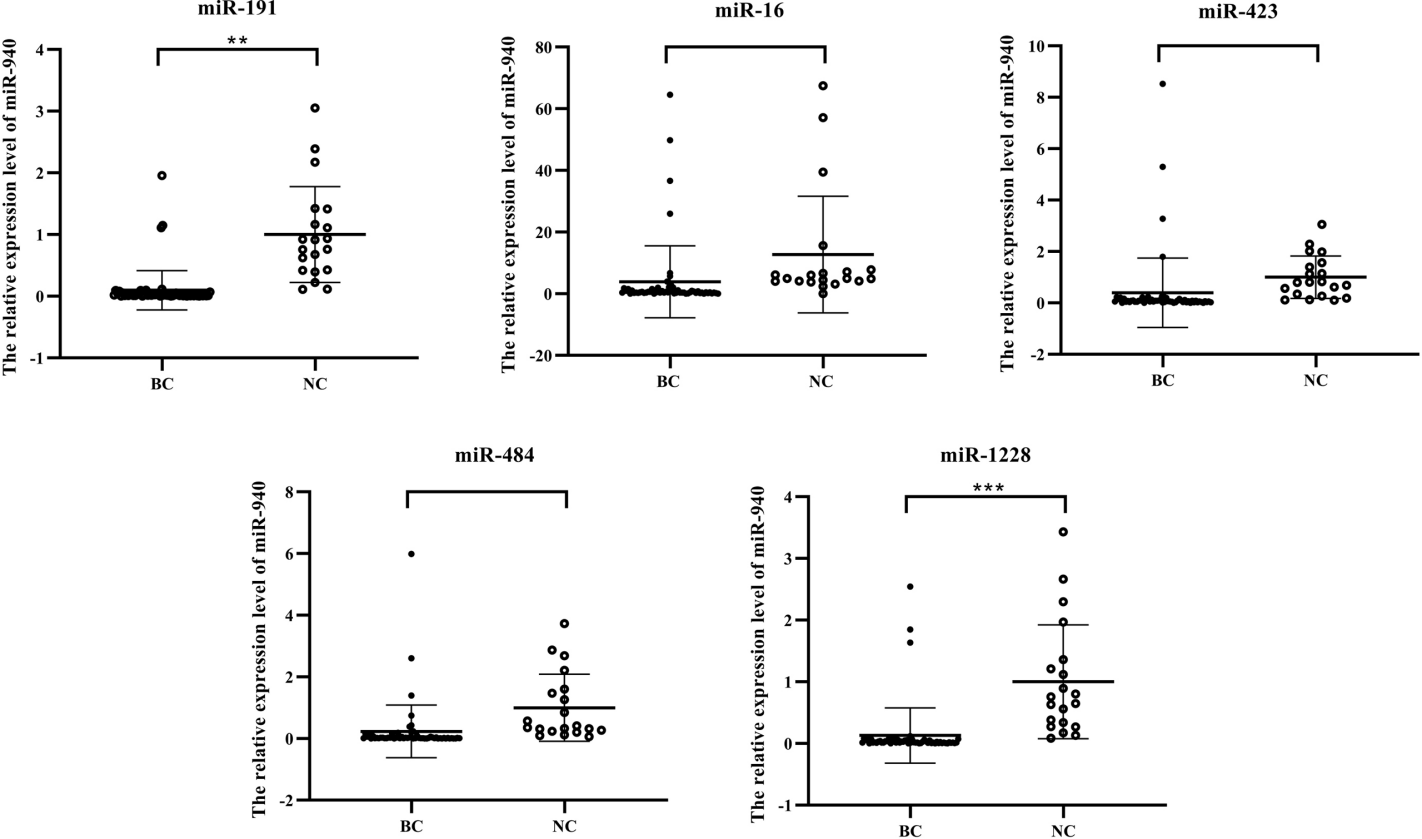
Results from normalization using the candidate internal reference miR-940. (*p<0.05, **p<0.01, ***p<0.001, no significance, ns p>0.05).

The expression level data of miR-940 were normalized (**Figure 4**) using the RefFinder program recommended for miR-1228, miR-191, and miR-423 and the geNorm program recommended for miR-484. Although miR-16 was not recommended as a suitable reference gene by BestKeeper and geNorm, we used miR-16 to normalize the content of miR-940 because this gene has often been used for expression studies (30, 31). When using different internal reference genes, the fold change in serum exosomal miR-940 in each group was calculated (**Figure 4**). The results from the normalization of miR-940 were used. miR-191 and miR-1228 showed that the serum exosomal miR-940 levels in breast cancer patients were significantly downregulated, whereas the results obtained by normalization using other candidate genes did not show the same result. This analysis showed that different normalization schemes may affect the quantitative expression of data. However, miR-940 should be assessed in a large sample study to confirm the reliability of the reference gene.

### Standard quantification of exosomal miR-940

**Figure 5:**
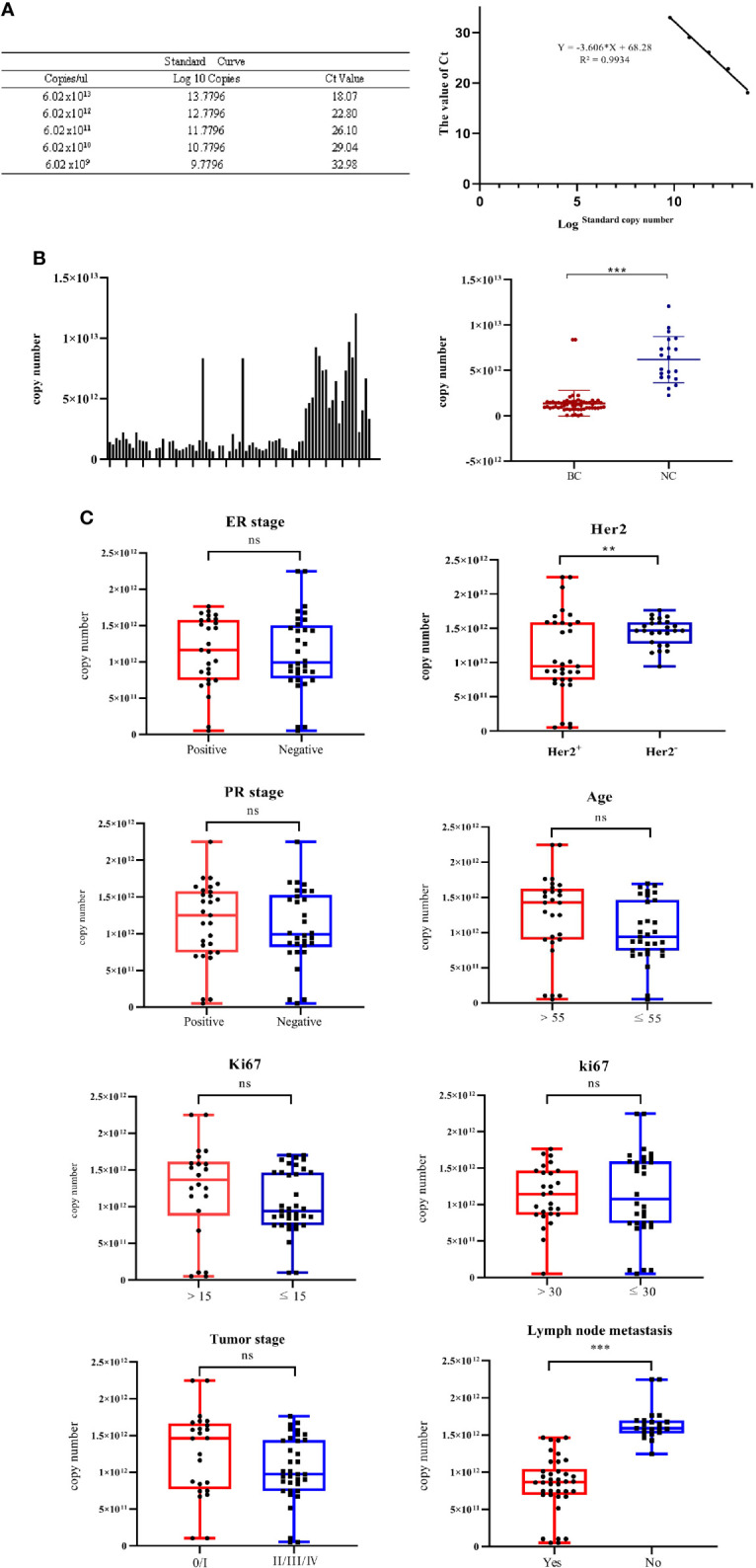
The correlations of the levels of exosomal miR-940 with a spectrum of pathophysiological parameters in cancer patients were tested. **(A)** Standard linear regression curve of miR-940. **(B)** The copy number of miR-940 transcripts per nanogram of exosomal RNA in each column sample, and the copy number of miR-940 in serum exosomes of breast cancer patients was significantly lower than that of normal controls. **(C)** Correlations of the levels of exosomal miR-940 with a spectrum of pathophysiological parameters in cancer patients. (*p<0.05, **p<0.01, ***p<0.001, no significance, ns p>0.05).

For the quantification of miR-940, a standard linear regression curve of the Ct values against the copy numbers was derived from serially diluted known amounts of miR-940 cDNA (**Figure 5A**). Based on the curve, the copy number of miR-940 transcripts per nanogram of exosomal RNA isolated from each cancer patient was determined. The copy number of miR-940 in serum exosomes of breast cancer patients was significantly lower than that of normal controls, which is similar to the results from the relative quantification of miR-940 using miR-191 and miR-1228 (**Figure 5B**), and this finding demonstrated that miR-191 and miR-1228 were appropriate reference genes. However, miR-16, miR-484 and miR-423 could not be used as reference genes for breast cancer exosomal miRNAs. These results indicated that the expression level of miR-940 in the serum exosomes of breast cancer patients was significantly downregulated.

To investigate the potential physiological significance of circulatory exosomal miR-940, the correlation of the levels of exosomal miR-940 with a spectrum of pathophysiological parameters in cancer patients was tested (**Figure 5C**). The patients were grouped according to various pathological indicators, and the grouping results are shown in **Table 5**. Among the 59 samples, we found that one of the breast cancer patients was male and the youngest, but we did not find any special pathological information, so we analyzed it in the same way. The patients were divided into two groups according to different parameters, and the corresponding miR-940 copy numbers were averaged within groups and compared with each other using a t test. The correlations between exosomal miR-940 and pathophysiological parameters in breast cancer patients are shown in **Figure 5C**, which showed that exosomal miR-940 levels were significantly lower in HER2/neu-positive patients than in HER2/neu-negative patients (median copy number: 9.43×10^11^ vs. 1.46×10^12^, P=0.017). The serum exosomal miR-940 levels were significantly higher in breast cancer patients without lymph node metastasis than in those with lymph node metastasis (median copy number: 8.7×10^11^ vs. 1.59×10^12^, P=1.110^-11^). However, the level of serum exosomal miR-940 was not related to the age, degree of differentiation, ER/PR status, Ki67 or TNM stage of the breast cancer patients (P>0.05). These results showed that the content of miR-940 in the serum exosomes of breast cancer patients was related to lymph node metastasis and HER2/neu expression status.

**Table 5:** Pathological grouping of blood samples.

Variable	Number	Variable	Number
age		ER	
≤55	28	(+)	27
>55	31	(-)	32
Lymph node metastasis		PR	
Negative	38	(+)	30
Positive	21	(-)	29
TNM stage		Ki67	
0/I	24	>15	32
II/III/IV	35	≤15	27
HER2		Ki67	
(+)	33	<30	37
(-)	26	≥30	22

## Discussion

With the development of precise tumor therapy and the continuous improvement of liquid biopsy technology, extracellular vesicles that can be secreted by various cells, such as exosomes, have attracted increasing attention. Exosomal miRNAs are not as degraded as free miRNAs in human fluids, and this type of RNA may be more suitable for the detection of tumor markers (32). Breast cancer is the leading cause of cancer death among women. Differences in the expression levels of circulating exosomal miRNAs between healthy people and breast cancer patients could identify molecular markers for the diagnosis and prediction of breast cancer (33). However, the detection methods of serum exosomal miRNAs in breast cancer patients have not been unified in clinical practice. Based on previous experiments, we first analyzed the effects of various exosome extraction methods and the starting amount of serum sample on miR-940, miR-16, miR-16 and miR-1228, analyzed the expression levels of three serum exosome miRNAs under hemolysis, and established a preprocessing method suitable for the clinical detection of serum exosomal miRNAs. To clinically detect the expression level of exosomal miRNA in breast cancer, an appropriate volume of insoluble blood samples should be used for serum exosomal RNA extraction using the membrane affinity method. We then demonstrated that miR-1228 and miR-191 could be used as reference genes for breast cancer serum exosomal miRNA, whereas miR-16, miR-484 and miR-423 were not suitable as reference genes. We also found that the expression level of serum exosomal miR-940 could reflect the presence of lymph node metastasis in breast cancer patients and the expression level of serum exosomal HER2/neu in breast cancer patients, indicating its potential as a metastasis marker.

As a novel diagnostic marker, exosomes have many advantages, such as their rapid, efficient and economic isolation and their important potential application value in clinical diagnosis and treatment (34). However, due to the complex formation environment and small diameter of exosomes, an ideal separation technology is key to limiting the research and application of exosomes. Among these, the preservation and pretreatment of samples is crucial. Ultracentrifugation was the first technique used for exosome isolation and remains the most common technique in articles (35). However, this method is time-consuming and complicated and requires a high amount of serum. Although precipitation can yield exosomes (36), the separated exosomes have very low purity because almost all soluble granules can settle, which is not conducive to downstream analysis. This study was used to extract exosomes using the membrane affinity column method with high efficiency and low loss obtained outside secreted RNA, which is suitable for clinical laboratory work and advantageous for downstream analysis (37). In addition, the storage state of serum samples is also very important in clinical testing, and hemolysis may occur. Studies have shown that the levels of various proteins, RNAs and DNAs in whole blood are greatly changed after hemolysis, whereas previous studies have shown that exosomal miRNAs are not as degraded as free miRNAs in body fluids. This study experimentally verified that hemolytic samples also have a great impact on the extraction of serum exosomal miRNA, which fills the gap in this aspect. Moreover, due to limited clinical serum resources, our study conducted a preliminary exploration of whether a sample size lower than that recommended by the protocol could achieve the same experimental effect.

Among the many techniques for the analysis of miRNA transcription levels, RT–qPCR is considered the most accurate technique due to its high sensitivity and easy reproducibility (38). Although relative quantification is a common method, it needs a reference gene for the normalization of different samples to ensure high sensitivity because the expression differences in the data results may not be due to the disease itself but rather to differences in the processes used for sample collection, stabilization, RNA extraction and target quantification. Therefore, identification of the best reference genes suitable for study is necessary for the accurate standardization of exosomal miRNA data. However, suitable reference genes for serum exosomal miRNAs in clinical breast cancer patients have not been reported to date. Based on previous reports, five candidate internal reference genes were selected for RT−qPCR studies of breast cancer using tissue, serum or plasma. Among them, miR-484, miR-423 and miR-1228 were used in the study of serum miRNA, and miR-16 and miR-191 were used in the study of breast tissue. Due to differences in the environment of exosomes, tissues and body fluids, the effect of these genes in standardizing breast cancer serum exosomes has been questioned. Therefore, we selected five genes as candidate genes to study their applicability as internal references for serum exosomal miRNAs in breast cancer patients. The results demonstrate that both miR-1228 and miR-191 could be used as reference genes for breast cancer serum exosomal miRNA. Although the results from the software analysis showed that miR-191 was not suitable as a reference gene for breast cancer serum exosomes, the relative quantification of the target genes using miR-191 also yielded the same results as those obtained with miR-1228. Similarly, high differences were observed when different miRNAs were used under the differentiation conditions described by Roulex-Bonin and Coste, which suggested that miR-191-5p was the most stable reference gene (39). Therefore, we believe that miR-191 could also serve as a suitable reference gene.

Previous studies have confirmed that the expression level of miR-940 in breast cancer tissues was significantly lower than that in adjacent tissues. Further studies found that miR-940 could inhibit the proliferation, invasion and migration of breast cancer cells by targeting and regulating CXC chemokine 2 (CXCR2) or ZNF24 (18, 40). Zhang et al. indicated that miR-940 induces malignant progression of breast cancer by regulating FOXO3 (41). In addition, the serum miR-940 levels in breast cancer patients predicted the efficacy of trastuzumab in patients with HER2-positive metastatic breast cancer (29). The downregulation of miR-940 levels in breast cancer tissues also led to the low content of free miR-940 in patient serum, and the stability of free miR-940 in serum was susceptible to environmental influences, which limited the ability of free miR-940 as a prognostic marker.

To verify whether breast cancer serum exosomal miR-940 has potential as a tumor marker of breast cancer, the copy number of miR-940 in the serum exosomes of each patient was calculated by an absolute quantitative method, and the results demonstrated that the content of miR-940 in the serum exosomes of breast cancer patients was significantly lower than that in normal human serum exosomes. The relationship between the copy number of serum exosomal miR-940 and the clinical data of breast cancer patients was analyzed.

The results showed that the expression level of miR-940 in the serum exosomes of breast cancer patients with lymph node metastasis was significantly downregulated. Therefore, the expression level of miR-940 in serum exosomes of patients can be used to judge whether a patient has lymph node metastasis. Similarly, we found that the expression level of miR-940 in the serum exosomes of HER2/neu-positive patients was significantly lower than that in those of HER2/neu-negative patients. However, as shown in **Figure 5C**, the differences were not as distinct due to the high dispersion of data points in both groups. Exosomal miR-940 alone may not be sufficiently accurate to judge the HER2/neu status of patients. We also demonstrated that serum exosomal miR-940 is a potential metastatic marker for breast cancer patients.

Li et al. found that exosomal miR-940 was mainly secreted by tumor cells *in vivo* through an analysis of exosomes and exosome-free supernatant from primary breast cancer cells and peripheral immune cells and revealed that miR-940 expression is increased in trastuzumab-sensitive HER2-positive metastatic breast cancer patients and further increased in trastuzumab-resistant patients (29). Therefore, they speculated that serum exosomal miR-940 has the potential to be used as an indicator of trastuzumab sensitivity in HER2/neu-positive metastatic breast cancer. Wang et al. found that the expression level of exosomal lncRNA-HOTAIR is able to reflect the HER2/neu status (21). Therefore, we hypothesized that the expression levels of exosomal miR-940 and lncRNA-HOTAIR may be used to judge the HER2/neu status of breast cancer patients. Of course, further experiments are needed to verify our hypothesis. In conclusion, serum exosomal miR-940 can be used as a minimally invasive liquid biopsy for monitoring disease progression.

In this study, we only selected some of the most frequently used reference genes in the literature and screened out the most stable reference genes. We did not sequence the RNA in the serum exosomes of breast cancer patients and normal people, and thus, more accurate reference genes may be obtained. When comparing clinical exosome extraction methods, the number of samples is small. If the sample size can be increased, the results may be more convincing. In addition, this paper describes one gene selected for miR-940, which shows that it can be used as a metastasis marker of breast cancer. We should test multiple miRNAs to increase its efficacy as a biomarker. Only one gene was selected. If multiple miRNAs are selected for combined detection with existing tumor markers, the reliability will be higher.

## Data availability statement

The raw data supporting the conclusions of this article will be made available by the authors, without undue reservation.

## Ethics statement

The studies involving human participants were reviewed and approved by The Ethics Committee of the Chongqing University Cancer Hospital. The patients/participants provided their written informed consent to participate in this study.

## Author contributions

ZG and HY performed and analyzed the experiments and wrote the manuscript. HWZ, HZ, and XHZ helped to perform the experiments. XDZ designed and supervised the study. All authors have read and approved the final manuscript.

## Funding

The present study was supported by a grant from the Fundamental Research Funds for the Central Universities (2019CDYGZD006).

## Acknowledgments

The authors of the present study are grateful for the valuable comments from members of the department of Breast Cancer, Chongqing University Cancer Hospital. The authors of this study also thank JunLi Huang from Chongqing University for valuable comments.

## Conflict of interest

The authors declare that the research was conducted in the absence of any commercial or financial relationships that could be construed as a potential conflict of interest.

## Publisher’s note

All claims expressed in this article are solely those of the authors and do not necessarily represent those of their affiliated organizations, or those of the publisher, the editors and the reviewers. Any product that may be evaluated in this article, or claim that may be made by its manufacturer, is not guaranteed or endorsed by the publisher.
